# Amphiphilic QP(DMAEMA-*co*-LMA)-*b*-POEGMA Random-Block Terpolymers as Nanocarriers for Insulin

**DOI:** 10.3390/biomedicines8100392

**Published:** 2020-10-04

**Authors:** Martha Kafetzi, Stergios Pispas, Xiaoyan Bao, Ping Yao

**Affiliations:** 1 Theoretical and Physical Chemistry Institute, National Hellenic Research Foundation, 48 Vassileos Constantinou Avenue, 11635 Athens, Greece; mkafetzi91@gmail.com; 2 Department of Macromolecular Science, Fudan University, 2005 Songhu Road, Shanghai 200438, China; 16110440009@fudan.edu.cn (X.B.); yaoping@fudan.edu.cn (P.Y.)

**Keywords:** random diblock terpolymer, polyelectrolytes, self-assembly, insulin, protein nanocarriers, protein delivery, polymer/insulin complexes, electrostatic interactions, in vitro cytotoxicity, in vivo biocompatibility

## Abstract

We report on the utilization of the amphiphilic poly[quaternized (2-(*N,N*-dimethylamino) ethyl methacrylate)]-*co*-(lauryl methacrylate))-*b*-poly[(oligo ethylene glycol) methyl ether methacrylate] QP(DMAEMA-*co*-LMA)-*b*-POEGMA cationic diblock terpolymer aggregates as nanocarriers for insulin delivery applications. QP(DMAEMA-*co*-LMA)-*b*-POEGMA random diblock terpolymer is derived from the chemical modification of the precursor amino diblock copolymer via quaternization, producing permanent positive charges on the macromolecular chain. The QP(DMAEMA-*co*-LMA)-*b*-POEGMA diblock terpolymer as well as its amino precursor investigated self-assemble in aqueous media, forming aggregates. In vitro cytotoxicity and in vivo biocompatibility studies on QP(DMAEMA-*co*-LMA)-*b*-POEGMA and its amino precursor aggregates, showed good cytocompatibility and biocompatibility. QP(DMAEMA-*co*-LMA)-*b*-POEGMA aggregates were chosen to be complexed with insulin due to their self-assembly features and the permanent positive charge in each amino group. QP(DMAEMA-*co*-LMA)-*b*-POEGMA aggregates were complexed with insulin through electrostatic interactions. Light scattering techniques were used in order to study the ability of the polymer aggregates to complex with insulin, to determine critical physicochemical parameters such as size, mass, and surface charge of the stable complexes and study the effect of salt addition on their properties. The results showed that in both cases, the complexation process was successful and as the insulin concentration increases, nanosized complexes of different physicochemical characteristics (mass, size, surface charge) and spherical morphology are formed. UV-Vis and fluorescence spectroscopy studies showed that no conformational changes of insulin occurred after the complexation.

## 1. Introduction

Over the last few years, protein and gene delivery have attracted significant scientific interest due to their promising application to modern therapeutic and diagnostic technologies to fight life-threatening diseases [1,2]. Cancer and diabetes mellitus are severe diseases that threaten a large percentage of the population worldwide [3,4]. Traditional medicinal practices, such as chemotherapy and insulin injections, respectively, are implemented to patients who suffer from these diseases. On the other hand, the side effects resulting from using such traditional tactics are sometimes severe and perilous for the human health. The discovery and implementation of nanotechnological approaches, more compatible to human body in order to diminish the side effects and offer to the patients a more convenient and less painful daily routine, is essential [5]. Nanotechnology-based drug and gene delivery are modern methods designed specifically for a better treatment of life-threating diseases and their beneficial characteristics over the traditional methods are unquestionable [5]. Nanocarriers are synthetic nanostructures that are able to encapsulate pharmaceutical molecules or complex with biomolecules such as DNA/RNA, proteins, liposomes etc., and deliver and release them to target cells without affecting the healthy tissues [6]. Their list of advantages is quite long and as a field that is currently developing, novel properties are still emerging. Nanocarriers combine significant physical and chemical properties that enhance the pharmacokinetics and biodistribution of pharmaceutical/biological molecules since they are capable of distributing the therapeutic substances with greater selectivity and specificity [7]. Moreover, the incorporation of the desired characteristics contributes to the high drug-loading efficacy and provides targeting of only damaged cells.

Diabetes is a high-risk metabolic disorder, affecting millions of people, in which the blood glycose levels are too high over a long period of time [8]. It is caused because either the pancreas is not able to produce the desired levels of insulin or the cells are resistant to insulin. In case the diabetes remains untreated, it can provoke many implications, such as cardiovascular diseases, stroke, and chronic kidney disease [9]. Consequently, patients suffering from Type-1 diabetes or Type-2 diabetes in later stages are forced to be injected with several doses of insulin every day, in order to keep their blood glycose concentration at regular levels [10].

Subsequently, research on insulin delivery should focus on the development of novel nanocarriers with the appropriate properties that will eventually enable the targeted complexation with insulin and its release as well as open other possible ways of administration. Block copolymers (BCPs) comprise a class of copolymers that consist of the repeat of several monomeric units in different, distinct blocks [11]. Monomers of different chemical properties can be combined in order to produce block copolymers of various architectures such as linear, star, hyperbranched, cyclic etc. [11]. The most attractive feature of the block copolymers is the ability to self-assemble in various media and subsequently to form nanostructures of controlled size, shape, and morphology. Those characteristics can be designed, controlled, and achieved via the synthesis of the block copolymer [12,13]. Block copolymers nanostructures are used extensively and with great success as nanocarriers for drugs and therapeutic biomacromolecules in defense of the human body against diseases. Block copolymer nanostructures prevail over other biomolecular carriers due to their small size, solubility in many solvents, colloidal stability, significant cellular uptake, high cell viability, and in vivo biocompatibility [14].

Cationic block polyelectrolytes have been implemented thoroughly for complexation and distribution of biomolecules such as nucleic acids and insulin [15]. The complexation is achieved through electrostatic interactions between the positive charges of the polyelectrolyte block and the negative charges of the biomolecule. The result of the complexation process is the formation of polyplexes of definite sizes, structures, and properties [16].

Specifically, poly[quaternized 2-(dimethylamino)ethyl methacrylate-*b*-lauryl methacrylate-*b*-(oligo ethylene glycol)methacrylate] (QPDMAEMA-*b*-PLMA-*b*-POEGMA) linear triblock terpolymers [17] and ABA-type triblocks such as PEO-*b*-PCL-*b*-PEO and PDMAEMA-*b*-PCL-*b*-PDMAEMA [18], numerous random [19], and diblock copolymers [20] have been utilized for insulin complexation/encapsulation and proposed for insulin treatment purposes, with some promising outcomes.

In this work, novel amphiphilic poly[quaternized (2-(*N,N*-dimethylamino) ethyl methacrylate)]-*co*-(lauryl methacrylate))-*b*-poly[(oligo ethylene glycol) methyl ether methacrylate] QP(DMAEMA-*co*-LMA)-*b*-POEGMA cationic block terpolymer aggregates were utilized for the complexation of insulin. To the best of our knowledge, terpolymers of such architecture, where the first block of a linear diblock terpolymer is composed of two randomly distributed monomeric units of different solubility in water, have not been reported before as insulin vectors. The complexation of the cationic diblock terpolymer aggregates was achieved via electrostatic interactions. The diblock terpolymers have permanent positive charges as a result of a chemical modification, while insulin at neutral pH values bears anionic charges. Hydrophobic interactions induced by the hydrophobic LMA segments, contribute to the formation of the complexes and define their physicochemical characteristics such as the size, stability, and further aggregation. The in vitro cytotoxicity and the in vivo biocompatibility of QP(DMAEMA-*co*-LMA)-*b*-POEGMA empty vectors were also studied. The complexation procedure and the resulted QP(DMAEMA-*co*-LMA)-*b*-POEGMA/insulin complexes were prepared via a simple-step protocol and investigated by light scattering methods (DLS, SLS, and ELS), fluorescence, UV-Vis, and ATR-FTIR spectroscopies.

## 2. Experimental Section

### 2.1. Chemicals and Reagents

**Scheme 1:**
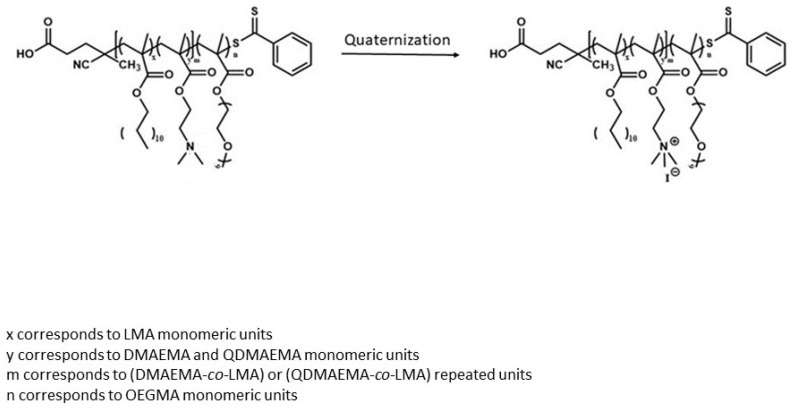
Chemical structures of P(DMAEMA-co-LMA)-b-POEGMA diblock terpolymer and its cationic analogue QP(DMAEMA-co-LMA)-b-POEGMA, as a result of quaternization process.

P(DMAEMA-*co*-LMA)-*b*-POEGMA diblock terpolymers precursors were prepared by implementation of two step reversible addition-fragmentation chain-transfer (RAFT) polymerization process, utilizing 4-cyano-4-(phenylcarbonothioylthio) pentanoic acid (CPAD) as the chain transfer agent (CTA). QP(DMAEMA-*co*-LMA)-*b*-POEGMA diblock terpolymers were obtained by performing a quaternization reaction to the P(DMAEMA-*co*-LMA)-*b*-POEGMA diblock terpolymers, using methyl iodide (CH_3_I) as the quaternizing agent. By carrying out the quaternization reaction, conversion of the tertiary amine groups of the PDMAEMA segments to quaternary, permanently positively charged QPDMAEMA units, was accomplished. The chemical structure of the QP(DMAEMA-*co*-LMA)-*b*-POEGMA diblock terpolymers utilized in this study is presented in Scheme 1.

The materials used, for the in vitro cytotoxicity studies of the unloaded QP(DMAEMA-co-LMA)-b-POEGMA aggregates include DMEM cell culture medium, fetal bovine serum, penicillin, and streptomycin, obtained from GIBCO BRL Life Technologies Inc. (Shanghai, China). MTS (3-(4,5-dimethylthiazol-2-yl)-5-(3-carboxymethoxyphenyl)-2-(4-sulfophenyl)-2H-tetrazolium) was from Promega Co. (Shanghai, China). HepG2 cell line (human liver carcinoma cells) was from American Type Culture Collection (ATCC, Manassas, VA, USA). For the in vivo biocompatibility studies of the neat QP(DMAEMA-*co*-LMA)-*b*-POEGMA aggregates, male ICR mice (25 ± 2 g) were from Sino-British SIPPR/BK Lab Animal Ltd. (Shanghai, China).

For the complexation of QP(DMAEMA-*co*-LMA)-*b*-POEGMA aggregates with insulin, recombinant human insulin of Mw = 5800 g∙mol^−1^ were purchased from Sigma-Aldrich (Athens, Greece) and utilized without further purification. Dimethyl sulfoxide (DMSO, ≥99.9%) and sodium chloride (NaCl, ≥99.0%) were also purchased from Sigma-Aldrich (Athens, Greece) and were used without further processing.

### 2.2. Preparation of QP(DMAEMA-co-LMA)-b-POEGMA/Insulin Polycomplexes

Firstly, two different concentration pairs of diblock terpolymers and insulin stock solutions were tested. The QP(DMAEMA-*co*-LMA)-*b*-POEGMA/insulin polyplexes were prepared by adding the appropriate quantities of insulin (dissolved in DMSO) dropwise to the aqueous solutions (0.01 M NaCl solution) of the terpolymers. The mixtures were kept under stirring for 20 min, at room temperature. Afterwards, the complexes were formed almost instantaneously. The mixing of the solutions was carried out according to calculations based on the charge ratios between insulin and the quaternary amino group of the terpolymers. Specifically, complexes of four different ratios were prepared and investigated. The (−)INS/(+)terpolymer ratios 0.25, 0.5, 0.75, and 1 were utilized. The Insulin is anionic at pH = 7 and contains four negative charges per molecule. In all cases, the total charge ratio of insulin is ≤1. The volume of the polymer that was added remained stable in every ratio, while different amounts of insulin volume were added in every case in order to get the appropriate charge ratio. The first concentration pair explored was c = 10^−3^ g·mL^−1^ for the stock solution of QP(DMAEMA-*co*-LMA)-*b*-POEGMA in 0.01M NaCl and c = 6 × 10^−3^ g·mL^−1^ for the insulin stock solution in DMSO. In every charge ratio, the volume of the polymer added was 2 mL, while 93 μL of the insulin stock solution were added in case the charge ratio is 0.25, 186 μL in case the charge ratio is 0.5, 278 μL in case the charge ratio is 0.75, and finally 371 μL in case the charge ratio is 1. The final volume of each mixture was 10 mL, so 0.01M NaCl was added in order to get the final volume. The final insulin concentrations, as the insulin charge increases by 0.25 were 5.6 × 10^−5^ g·mL^−1^, 1.12 × 10^−4^ g·mL^−1^, 1.67 × 10^−4^ g·mL^−1^ and 2.3 × 10^−4^ g·mL^−1^. The final polymer solution was 2 × 10^−4^ g·mL^−1^. The second concentration pair was c = 10^−4^ g·mL^−1^ for the stock solution of QP(DMAEMA-*co*-LMA)-*b*-POEGMA in 0.01 M NaCl and c = 4 × 10^−4^ g·mL^−1^ for the insulin stock solution in DMSO. In every charge ratio, the volume of the polymer added was 1 mL, while 62.5 μL of the insulin stock solution were added in case the charge ratio is 0.25, 125 μL in case the charge ratio is 0.5, 187.5 μL in case the charge ratio is 0.75, and finally 250 μL in case the charge ratio is 1. The final insulin concentrations, as the insulin charge increases by 0.25 were 5 × 10^−6^ g·mL^−1^, 1 × 10^−5^ g·mL^−1^, 1.5 × 10^−5^ g·mL^−1^, and 2 × 10^−5^ g·mL^−1^. The final polymer solution was 2 × 10^−5^ g·mL^−1^. The final volume of each mixture was 5 mL, so 0.01 M NaCl was added in order to get the final volume. Different concentrations have been utilized in order to get complexes of different structural characteristics and check their colloidal stability.

### 2.3. Methods

#### 2.3.1. In Vitro Cytotoxicity Assays

In vitro cytotoxicity of the precursor P(DMAEMA-*co*-LMA)-*b*-POEGMA and the modified QP(DMAEMA-*co*-LMA)-*b*-POEGMA were determined by MTS assay. HepG2 cells were seeded in 96-well plate at a density of 5 × 10^3^ cells/well. The cells were incubated in complete culture medium (DMEM medium containing 10 v% fetal bovine serum, 100 IU.mL^−1^ penicillin and 100 μg·mL^−1^ streptomycin) at 37 °C in a cell incubator under humid atmosphere containing 5% CO_2_. After 24 h incubation, the cells were treated with fresh medium containing P(DMAEMA-*co*-LMA)-*b*-POEGMA or QP(DMAEMA-*co*-LMA)-*b*-POEGMA at the terpolymer concentration of 0, 31.2, 62.5, 125, 250, or 500 μg·mL^−1^ in triplicate (*n* = 3). After 48 h of incubation, the cell media were replaced by 120 μL fresh medium containing 16.7 v% MTS and the cells were incubated for another 2 h in the dark. Then, the 490 nm absorbance of the plate was read on a BioTek microplate reader (Winooski, VT, USA). The cell viability was calculated using the following equation:

Cell viability (%) = (A_Sample − A_MTS)/(A_Blank − A_MTS) × 100%(1)

where A_Sample was the absorbance of the cells after the polymer treatment, A_blank was the absorbance of the cells without the polymer treatment, and A_MTS was the absorbance of the complete culture medium containing MTS.

#### 2.3.2. In Vivo Biocompatibility Assays

In vivo assays were performed for P(DMAEMA-*co*-LMA)-*b*-POEGMA and the QP(DMAEMA-*co*-LMA)-*b*-POEGMA. The animal experiments of this study were performed at the Experimental Animal Center of School of Pharmacy of Fudan University in full compliance with the guidelines approved by Shanghai Administration of Experimental Animals. Healthy mice were, respectively, injected with P(DMAEMA-*co*-LMA)-*b*-POEGMA or QP(DMAEMA-*co*-LMA)-*b*-POEGMA solution subcutaneously at 1 mg·kg^−1^ dose once daily. Saline solution (0.9% NaCl) was injected subcutaneously as a control. After 15 days of continuous injections, the mice were sacrificed. The organs of heart, liver, spleen, lung, and kidney were surgically taken out, fixed, dehydrated, and embedded in paraffin in succession. The specimens were cut into 5 μm thick sections, and the sections were stained with hematoxylin-eosin. The hematoxylin−eosin stained histological images of the sections were observed on a microscope (BX53, OLYMPUS, Tokyo, Japan).

#### 2.3.3. Light Scattering Investigations

Dynamic light scattering measurements were carried out on an ALV/CGS-3 compact goniometer system (ALVGmbH, Hessen, Germany), connected with an ALV 5000/EPP multi-τ digital correlator with 288 channels and an ALV/LSE-5003 light-scattering electronics unit for stepper motor drive and limit switch control. A JDS Uniphase 22 mW He-Ne laser (λ = 632.8 nm) was utilized as the light source. Measurements of the intensity correlation function were carried out five times for each concentration and angle and were averaged for each angle. The solutions were filtered through 0.45 μm hydrophilic PVDF filters (Millipore, Billerica, MA, USA) before measurements. The angular range for the measurements was 30–150°. Obtained correlation functions were analyzed by the cumulants method and the CONTIN software (ALVGmbH, Hessen, Germany). The size data and figures depicted below are from measurements at 90°.

Static light scattering (SLS) experiments were conducted on the same instrument at angles 30–150°. Toluene was used as the calibration standard. The R_g_/R_h0_ ratios were calculated by implementing the Guinier, second order method.

For the ionic strength dependent light scattering measurements, the ionic strength of the QP(DMAEMA-*co*-LMA)-*b*-POEGMA/INS solution was increased by gradual addition of the appropriate volume of a 1 M NaCl stock solution). After each addition, the solution was stirred and left to equilibrate for 15 min before measurement.

Electrophoretic light scattering (ELS) studies were conducted on a ZetaSizer Nano series Nano ZS (Malvern Instruments Ltd., Malvern, Worcestershire, UK), composed of an He-Ne laser at a wavelength of 633 nm and a fixed backscattering angle of 173°. The Henry correction of the Smoluchowski equation was implemented to analyze the obtained data, after equilibration at 25 °C. The recorded zeta-potential values are averages of 100 scans.

#### 2.3.4. UV-Vis Investigations

Optical absorption spectra of the QP(DMAEMA-*co*-LMA)-*b*-POEGMA/INS mixed solutions were recorded on a Perkin–Elmer, Lambda 19 spectrophotometer. The measurements were carried out in the range of 200–400 nm (UV range).

#### 2.3.5. Fluorescence Spectroscopy Investigations

Fluorescence spectra were recorded on a Fluorolog-3 JobinYvon-Spex spectrofluorometer (model GL3–21, Kyoto, Japan) in the range between 300 and 500 nm. The excitation wavelength for the measurements was 280 nm. In this way the intrinsic fluorescence of phenylalanine residues of INS is probed and used to verify conformational changes of insulin in the complexes.

#### 2.3.6. FTIR-ATR Spectroscopy Investigations

FTIR-ATR measurements were conducted at room temperature in the spectral range 5000 to 500 cm^−1^ on a Bruker Equinox 55 Fourier transform instrument, equipped with a single bounce attenuated total reflectance (ATR) diamond accessory (Dura-Samp1IR II by SensIR Technologies). Background spectrum was collected by recording the clean and dry ATR diamond crystal surface against air and then utilizing its value to be subtracted from the sample spectrum. The experiments were performed in the dry state after solvent evaporation. Nitrogen was used in order to dry the solutions and finally receive thin films of the complexes. Additionally, 64 interferograms were collected for every single spectrum with a resolution of 4 cm^−1^ to obtain the better signal/noise ratio.

#### 2.3.7. Size Exclusion Chromatography Investigations

For the determination of the molecular weight and the molecular weight distribution of the synthesized P(DMAEMA-co-LMA)-b-POEGMA diblock copolymer, a Waters SEC instrument was used. It was set up with a Waters 1515 isocratic pump, a set of three μ-Styragel mixed bed columns (pore diameter between 10^2^ and 10^6^ Å), and a Waters 2414 refractive index detector (equilibrated at 40 °C). Breeze software was employed for data acquisition and analysis. THF (containing 5% *v/v* triethylamine) consisted the mobile phase, at a flow rate of 1.0 mL/min, at 30 °C. Polystyrene standards of average molecular weights between 1200 and 152,000 g/mol and narrow molecular weight distributions were utilized for the calibration of the SEC set-up. In order to determine the molecular weights and the molecular weight distributions, the copolymers were dissolved in THF at concentrations of 2–4 g/mL. The results obtained from SEC analysis are presented in Table 1.

#### 2.3.8. ^1^H-NMR Investigations

^1^H-NMR experiments were conducted on a Varian 300 (300MHz) spectrometer using Vjnmr software and tetramethylsilane (TMS) as the internal standard in D_2_O. ^1^H-NMR spectroscopy was employed for obtain the actual weight composition (%wt) of both P(DMAEMA-*co*-LMA)-b-POEGMA and QP(DMAEMA-*co*-LMA)-b-POEGMA diblock terpolymers. The calculations were done using characteristic peaks for each type of segments in the spectra. The determined weight fractions of each segment are presented in Table 1.

**Figure 1:**
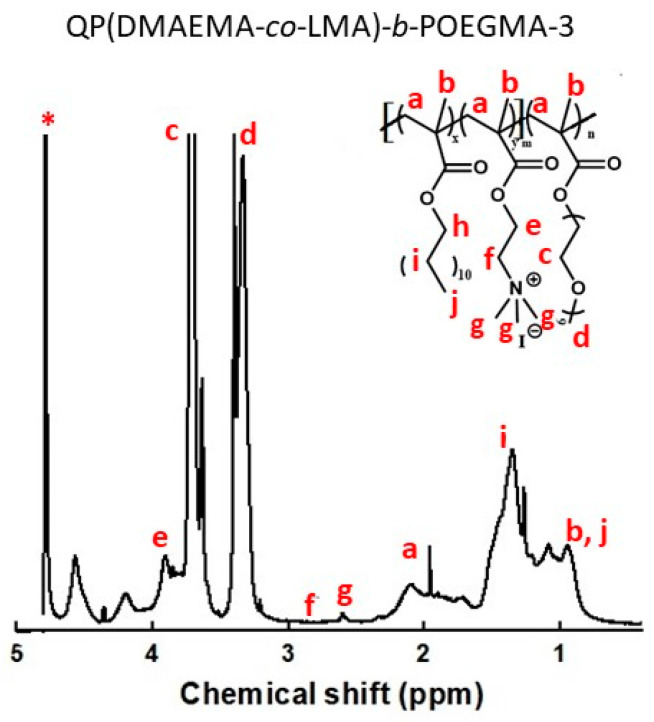
^1^H-NMR spectrum of the QP(DMAEMA-co-LMA)-b-POEGMA-3 in D_2_O. Asterisk denotes solvent (D_2_O) peak.

^1^H-NMR peaks of the QP(DMAEMA-*co*-LMA)-*b*-POEGMA-3, (Figure 1, 300 MHZ, D_2_O, ppm): 4.1 (2H, - COOC*H_2_* (CH_2_)_10_CH_3_-) 3.82 (4H, -(C*H_2_*C*H_2_*O)_9_CH_3_-), 3.41 (3H, -(C*H*_2_C*H*_2_O)_9_CH*_3_*-), 3.02 (2H, -OC*H_2_*C*H_2_*NH(CH_3_)_2_-), 2.72 (9H, -NH(C*H_3_*)*_3_*-), 2.21 (2H, -C*H_2_*C-), 1.35 (2H, -CH_2_(CH*_2_*)*_10_*CH_3_-), 0.98 (3H, -CH_2_CC*H_3_*-, 3H, -CH_2_(CH_2_)_10_C*H_3_*).

## 3. Results and Discussion

The ability of the QP(DMAEMA-*co*-LMA)-*b*-POEGMA cationic diblock terpolymer aggregates to complex with insulin and form complexes is examined. In order to understand in depth and compare the changes in size, morphology, and conformation that occur during the complexation process, the molecular and self-assembly behavior characteristics of the P(DMAEMA-*co*-LMA)-*b*-POEGMA and QP(DMAEMA-*co*-LMA)-*b*-POEGMA unloaded vectors are xmentioned briefly. The molecular and self-assembly behavior experimental results were determined by applying SEC, ^1^H-NMR, and LS techniques. As an example, the ^1^H-NMR spectrum of the QP(DMAEMA-*co*-LMA)-*b*-POEGMA-3, is presented. The successful transformation of the tertiary amino group into quaternary was verified by ^1^H-NMR (Figure 1). The appearance of a new peak at 2.68 ppm assigned to the protons of the quaternary amine groups (9H, –N^+^(CH_3_)_3_) and the disappearance of the peak attributed to the protons of the tertiary amine groups confirm that the quaternization reaction was, indeed, quantitative. The assignment of the protons of each group to the characteristic spectral peak can be observed in Figure 1. The composition of the diblock terpolymers copolymers was calculated using the characteristic spectral peaks of the QPDMAEMA at 2.68 ppm, of PLMA at 1.35 ppm and of POEGMA at 3.82 ppm.

Below, in Table 1, the molecular characteristics and self-assembly properties (experimental results) of the diblock terpolymers, are presented.

By implementing the RAFT polymerization process, the preparation of the desired P(DMAEMA-*co*-LMA)-*b*-POEGMA diblock terpolymer, was accomplished. Afterwards, by quaternizing the random block, the desired QP(DMAEMA-*co*-LMA)-*b*-POEGMA cationic diblock terpolymer was obtained. Their molecular characteristics were the expected ones. The composition ratios were almost identical to the calculated ones. Subsequently, the synthesized diblock terpolymers could be tested for their ability to self-assemble, as well as for their cytotoxicity, in vivo biocompatibility and possible application of the quaternized diblock terpolymer aggregates, as insulin nanocarriers. Based on DLS results, presented in Table 1, the P(DMAEMA-*co*-LMA)-*b*-POEGMA diblock terpolymer self-assemble when is inserted in aqueous media (0.01 M NaCl), forming two types of particles. It is expected that copolymers containing DMAEMA groups respond to variation of solution pH and so the adjustment between protonated-deprotonated phases of the amino groups affects the solubility of the copolymer in aqueous media and the formation of multimolecular nanoparticles. The self-assembly ability, observed by DLS experiments, was studied in at pH = 7 where tertiary amine groups of DMAEMA monomeric units are partially protonated. Based on the respective sizes the first population (at R_h_~3 nm) is attributed to single polymer chains, which are molecularly dissolved in the aqueous medium. The second one is nanoaggregates from the aggregation of several diblock chains. The concurrence of unimers and aggregates derives from both the partial protonation of the amine groups of the DMAEMA segments and the existence of the hydrophobic LMA segments. Obviously, the hydrophobic interactions between LMA segments are responsible for the formation of nanoaggregates. Apparently, not all macromolecular chains participate to the nanoaggregates. The positive but not far from zero, zeta-potential values are expected, recalling that the aggregates were formed at pH = 7, were the amino groups are partially protonated (and positively charged). When the quaternization reaction takes place, the DMAEMA segments are converted into strong cationic segments [21]. The positive charges are permanent irrespective of the solution pH. Based on DLS, only one kind of aggregate is formed in QP(DMAEMA-*co*-LMA)-*b*-POEGMA aqueous solutions presenting smaller size than the non-quaternized precursors. Τhis fact is attributed to the increased solubility of the macromolecular chains and the formed nanostructures arise exclusively from hydrophobic interactions, developed between LMA segments. Considering, the small hydrophobic LMA content of the diblocks the formation of aggregates of spherical geometry is anticipated. In such nanoassemblies the core of the aggregates should be composed of the QP(DMAEMA-co-LMA) quaternized random blocks, which is assumed to be of a mixed polymeric nature and may contain some water molecules due to the hydrophilicity of QDMAEMA segments.

As anticipated, the aggregates display strong positive zeta-potential values due to the positively charged ammonium group of QDMAEMA segments.

**Figure 2:**
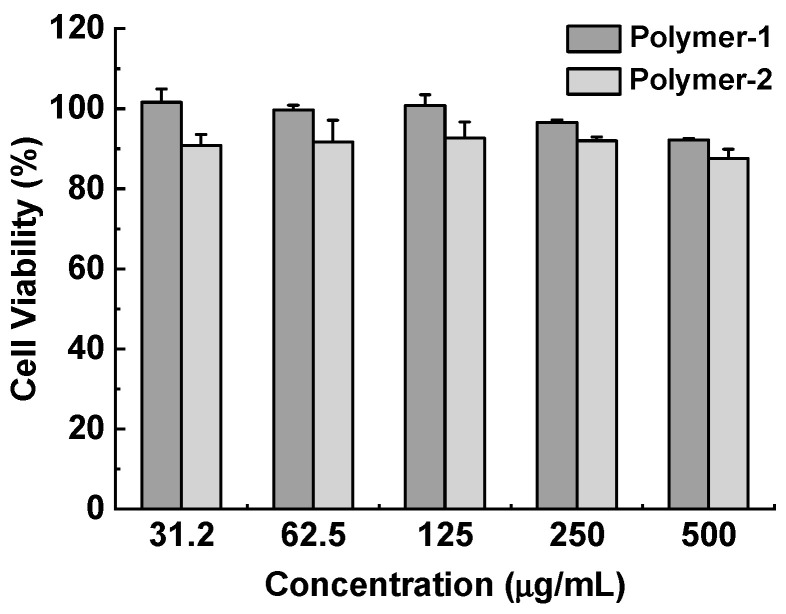
HepG2 cell viability after 48 h incubation with P(DMAEMA-co-LMA)-b-POEGMA (Polymer-1) and QP(DMAEMA-co-LMA)-b-POEGMA (Polymer-2) separately at different polymer concentrations (*n* = 3).

Cytotoxicity assays were conducted on the aggregates of the P(DMAEMA-*co*-LMA)-*b*-POEGMA and QP(DMAEMA-*co*-LMA)-*b*-POEGMA in order to determine whether these diblock terpolymers meet the necessary biological criteria to be utilized as insulin carriers. Endogenous insulin mainly takes effect in the liver [22,23]. The HepG2 cell viability results are depicted in Figure 2. For brevity, P(DMAEMA-*co*-LMA)-*b*-POEGMA aggregates will be referred as Polymer-1 and QP(DMAEMA-*co*-LMA)-*b*-POEGMA aggregates as Polymer-2, respectively.

Figure 2 demonstrates that after 48 h incubation at the polymer concentration of 500 μg·mL^−1^, the cell viability was higher than 85%, compared with the cell viability without the polymer treatment, verifying that both polymers exhibit significant biocompatibility. In fact, as it was expected Polymer-1 displays higher biocompatibility than Polymer-2. PDMAEMA contains solely tertiary amino groups and its transfection efficiency attains 90% of the one that polyethylenimine (PEI) exhibits [24]. On the other hand, by quaternizing the DMAEMA segments even though the polymer hydrophilicity is enhanced the cytotoxicity is expected to be higher, probably due to the permanent positive charges at the quaternary amino groups. Our case seems to follow that perspective but the results are really encouraging for both polymers investigated and especially for the aggregates of the quaternized analogue that is expected to form more stable electrostatic complexes with biomolecules due to the positive charges that enhance the complexation process. To sum up, the high biocompatibility that both terpolymers exhibit, indicates that they are both promising candidates as insulin nanocarriers.

**Figure 3:**
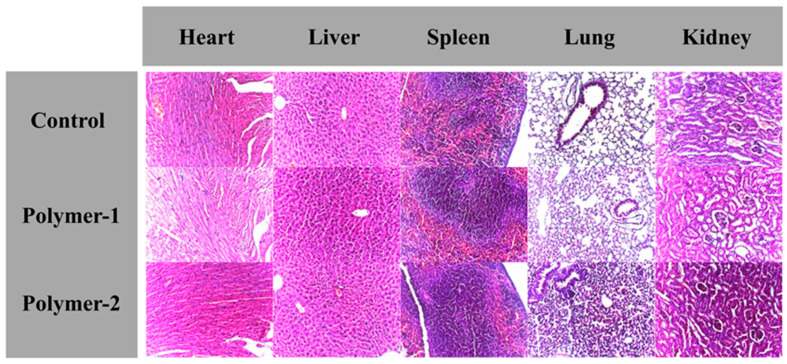
Representative hematoxylin−eosin stained histological images of the organs excised after subcutaneous injections with P(DMAEMA-co-LMA)-b-POEGMA (Polymer-1) and QP(DMAEMA-co-LMA)-b-POEGMA (Polymer-2) separately at a dose of 1 mg/kg for 15 days continuously. Saline solution (0.9% NaCl) was injected subcutaneously as control. The images were acquired at a magnification ratio of 100.

As the cell viability assays were promising, in vivo biocompatibility studies, for both terpolymers, were performed. Solutions of both polymers were injected subcutaneously in healthy mice separately at a dose of 1 mg·kg^−1^ for 15 days continuously. The hematoxylin−eosin stained histological images of the murine organs are shown in Figure 3.

Τhe histological images of the heart, liver, spleen, lung, and kidney organs, show no significant morphological difference in the tissues, after the solutions of P(DMAEMA-*co*-LMA)-*b*-POEGMA and QP(DMAEMA-*co*-LMA)-*b*-POEGMA terpolymers were injected for 15 days continuously, compared with the control group. This fact indicates that the two polymers are indeed in vivo biocompatible.

After verifying that the P(DMAEMA-*co*-LMA)-*b*-POEGMA and QP(DMAEMA-*co*-LMA)-*b*-POEGMA aggregates present low cytotoxicity and high in vivo biocompatibility, physicochemical studies were performed in respect to the complexation between the Q(PDMAEMA-*co*-LMA)-*b*-POEGMA aggregates and insulin. The experiments concern only the quaternized analogue, as the permanently positive charges would provide the formation of complexes of greater stability.

**Scheme 2:**
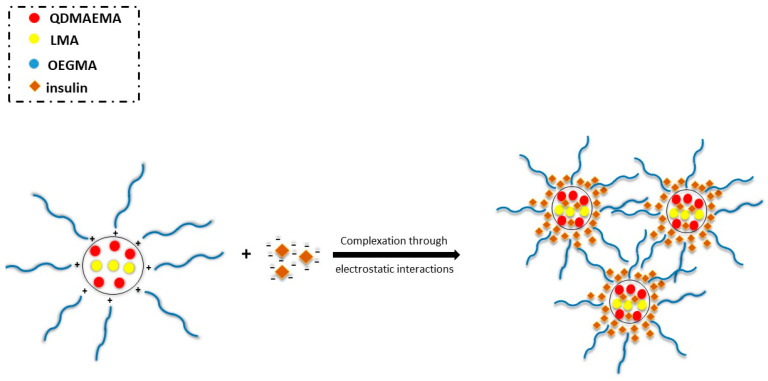
Schematic illustration of the complexation between QP(DMAEMA-co-LMA)-b-POEGMA aggregates and insulin.

As it was mentioned before, the complexation results from the electrostatic interactions between the positive charges of the quaternary ammonium salt groups of the polymer and the negative charges that insulin molecules contain at pH = 7. In this case, based on the molecular characteristics of the quaternized diblock terpolymer, ten positive charges correspond to each macromolecular chain, while each insulin molecule contains four negative charges [22]. A schematic demonstration of the polyplexes formed as the result of the complexation of the QP(DMAEMA-*co*-LMA)-*b*-POEGMA aggregates with insulin, is presented in Scheme 2. In this case, insulin molecules are anticipated to be complexed with the QPDMAEMA segments, which constitute the greatest part of the core due to polymer composition, but it is also expected that the QPDMAEMA segments would comprise mostly the periphery/external part of the core and the interface between core and corona, due to their hydrophilic nature. LMA segments would occupy the internal part of the core due to hydrophobic interactions, which results in the formation of stable QP(DMAEMA-*co*-LMA)-*b*-POEGMA/insulin complexes. POEGMA neutral hydrophilic macromolecular chains constitute the corona of the mixed complexes and contribute significantly to the colloidal stabilization of the complexes in the aqueous medium.

Size, scattered intensity, surface charge, and R_g_/R_h0_ ratio are significant parameters for determining whether the complexation of the polymeric nanoaggregates with insulin molecules was achieved. The values of these parameters are able to confirm the ability of QP(DMAEMA-*co*-LMA)-*b*-POEGMA aggregates to act as insulin nanocarriers. Hence, dynamic, static and electrophoretic light scattering measurements were conducted to determine the above features of the resulting polymer/protein complexes. The studies were carried out by utilizing polymer and insulin stock solutions of two different concentrations. In both cases, the protocol that was followed for the preparation of the complexes, was the same. The experiments performed in these two different cases, aim at determining the most appropriate conditions in terms of size, surface charge, and colloidal stability of the formed complexes over time. It is very important to note that in both cases during and after the complexation process as well as the next day mixing the stock solutions, no precipitation phenomena were observed. This fact may be attributed at least partially to the polymer architecture (in particular the random mixing of hydrophilic and hydrophobic segments in one of the blocks) and the presence of the LMA segments that induce hydrophobic interactions and provide structural integrity to the mixed nanosystem, while colloidal stability if provided by the POEGMA blocks.

**Figure 4:**
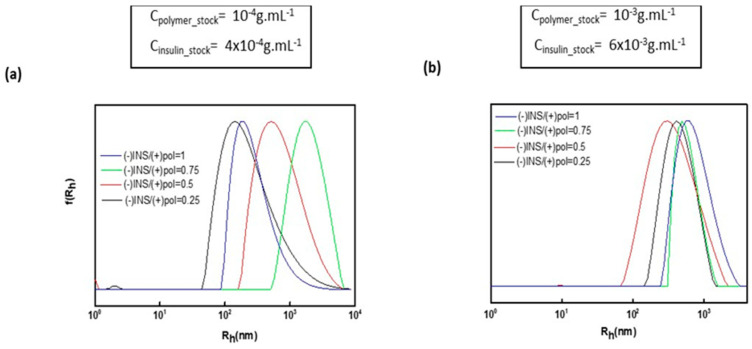
Size distributions from CONTIN analysis for the QP(DMAEMA-co-LMA)-b-POEGMA/insulin complexes when (**a**) the concentration of the polymer stock solution is 10*^−^*^4^ g·mL*^−^*^1^ and the insulin stock solution is 10*^−^*^4^ g·mL*^−^*^1^, (**b**) the concentration of the polymer stock solution is 10*^−^*^3^ g·mL*^−^*^1^and the insulin stock solution is 6 × 10*^−^*^3^ g·mL*^−^*^1^.

In Figure 4, size distributions from CONTIN analysis of the polymer/insulin complexes, obtained from DLS measurements, are displayed. For size comparison reasons, size distribution graphs for different stock concentrations are shown. It is observed that in both cases, in each (−)INS/(+)polymer charge ratio, a monomodal peak is depicted, indicating that only one type of complexed species is present in the aqueous medium. This information is of major importance, as we are able to deduce that only complexed macromolecules exist in the solution and no free/uncomplexed diblock terpolymer aggregates and uncomplexed insulin molecules can be detected. In addition, in both cases, the (−)INS/(+)polymer charge ratio plays a significant role in the size of the complexes, as in each case complexes of different size are formed. In particular, in the case where the concentration of polymer stock solution is 10^−4^ g·mL^−1^, the complexes that are formed are of larger size than the ones where the polymer stock solution is 10^−3^ g·mL^−1^, presumably because more H_2_O molecules exist in the inner part of the mixed particles. In Figure 4a, it is observed that the size depends on the insulin content. Specifically, as the concentration of insulin increases, the size of the complexes also increases, except when the (−)INS/(+)polymer is 1, where the size of complexes decreases. This fact is probably interpreted by the existence of a larger number of insulin molecules (i.e., negative charges) that leads to the participation of more polymer chains (of positive charge) in the complexes. In the second case, a small tendency towards a size increase as the insulin content increases is observed, except when the (−)INS/(+)polymer charge ratio is 0.5. However, the dimensions do not differ remarkably.

**Figure 5:**
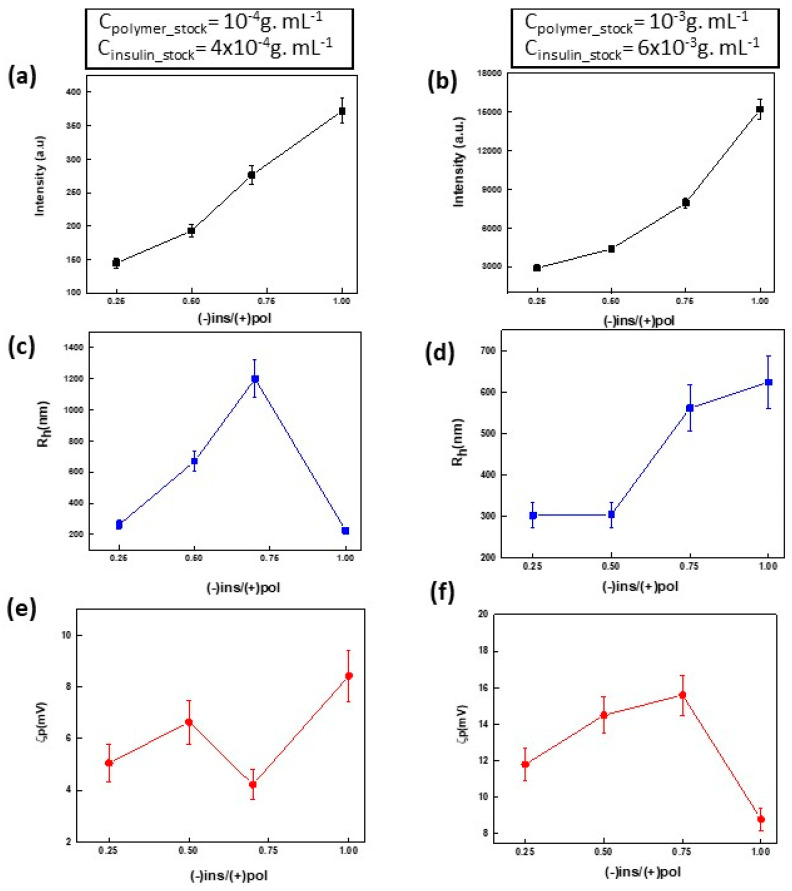
(**a**) Intensity, (**c**) R_h_, (**e**) zeta potential (surface charge) plots as a function of (−)INS/(+)polymer charge ratio for the case of the polymer stock solution with concentration c = 10*^−^*^4^ g·mL*^−^*^1^ and of insulin stock solution c = 4 × 10*^−^*^4^ g·mL*^−^*^1^, and (**b**) intensity, (**d**) R_h_, (**f**) zeta potential (surface charge) plots as a function of (−)INS/(+)polymer charge ratio for the case of the polymer stock solution concentration c = 10*^−^*^3^ g·mL*^−^*^1^ and of the insulin stock solution c = 6 × 10*^−^*^3^ g·mL*^−^*^1^.

In Figure 5, the scattering intensity, hydrodynamic radius, and surface charge as a function of (−)INS/(+)polymer charge ratios, for both cases of stock concentrations, are presented. Concerning the scattering intensity, obtained from DLS studies, the same tendency, in both concentration cases, is observed. As the insulin content increases, the scattering intensity also increases. This means that complexes of higher mass are formed, as more insulin is added to the mixtures. However, the complexes, derived from mixing the polymer stock solution of concentration c = 10^−3^ g·mL^−1^ with the insulin stock solution of c = 6 × 10^−3^ g·mL^−1^, are of higher mass compared with the ones derived from mixing the other concentration pair (scattering intensity values are between 2300–15800 a.u/Figure 5b). The scattering intensity values, for the case where the polymer stock solution is 10^−4^ g·mL^−1^, fluctuate within 150–380 a.u/Figure 4a. Combined with the information gained from the behavior of hydrodynamic radius, as a function of (−)INS/(+)polymer charge ratio, we conclude that when the polymer stock solution of 10^−4^ g·mL^−1^ is used, complexes of larger dimensions but of smaller mass, are formed. This practically means that the lower concentrations of macromolecules lead to more swelled complexes. In the first case (c_polymer_stock_=10^−4^ g·mL^−1^), the hydrodynamic radius values are between 240 and 1100 nm (Figure 5c), and as insulin concentration increases, the size of the complexes increases rapidly until the (−)INS/(+)polymer charge ratio is equal to 1. In this charge ratio, the dimensions of the complexes (R_h_~240 nm) are reduced compared with the two previous ones, indicating that when the negative charges from insulin in the solution increase, more positively-charged polymer chains participate in the complexation process, leading to tighter and of higher mass complexes. In the second case (c_polymer_stock_=10^−3^ g·mL^−1^), the hydrodynamic radius exhibits the same upward tendency as the scattering intensity, suggesting that as the insulin concentration in the solution rises, more polymer chains interact electrostatically with them, forming complexes of both increased mass and size dimensions. The hydrodynamic radius values fluctuate between 300 and 630 nm (Figure 5d). It should be noted that in both cases the sizes of the complexes are larger than that of the polymeric aggregates. This proves that, in fact, the formation of the QP(DMAEMA-*co*-LMA)-*b*-POEGMA/insulin complex is successful and the complexes are comprised of several polymer aggregates “glued together” by insulin molecules. The zeta potential values (i.e., the surface charge), obtained from ELS measurements (Figure 5e,f), are positive but not of appreciable difference, in both cases of stock solutions concentrations. This observation may prove the possibility of the formation of aggregates of complexes, which also supports the hypothesis that the formed complexes obtain an internal structure in which the cationic segments should occupy the perimeter of the complexes, while the insulin molecules are hidden in the internal part of the complexed aggregates. The highest ζp positive values are observed in cases where the polymer concentration is 10^−3^ g·mL^−1^, and they are correlated to the higher polymer concentration. Apparently, as the number of polymer molecules increases more positive charges exist in the periphery/surface of the complex.

R_g_/R_h_ values from SLS measurements in the case of the polymer stock concentration of 10^−4^ g·mL^−1^ are found in the range from 0.25 to 0.5, indicating that the formed complexes acquire spherical geometry with a rather loose structure.

Even though, the results obtained from the light scattering techniques were more encouraging for the case of polymer stock solution concentration equal to 10^−3^ g·mL^−1^, the third day after the complexes were prepared, precipitation phenomena occurred at all ratios. Subsequently, either the insulin or the polymer stock solution concentration was too high for the complexes to remain stable and dispersed in the aqueous medium. Further studies of the complexes were conducted only to the first case (c_polymer_stock_ = 10^−4^ g·mL^−1^, c_insulin_stock_ = 4 × 10^−4^ g·mL^−1^), where the complexes remained stable even after weeks from the day they were prepared. Apparently, concentration of the stock solutions also influences the stability of the formed polymer/protein complexes.

**Figure 6:**
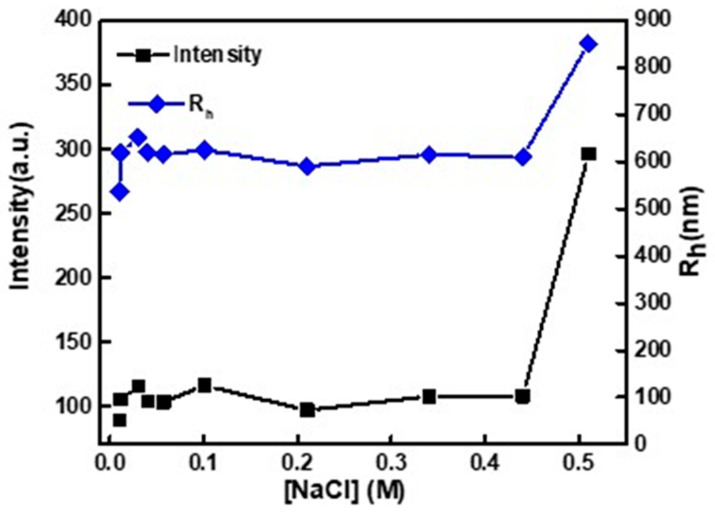
Scattering intensity and R_h_ as a function of ionic strength for the case where the polymer stock solution concentration is 10*^−^*^4^ g·mL*^−^*^1^ and the (−)INS/(+)polymer ratio is equal to 0.25.

Ionic strength studies were performed in the mixed solution where the (−)INS/(+)polymer ratio was equal to 0.25. In Figure 6, variations of scattering intensity and R_h_ as a function of ionic strength are depicted. Ionic strength is a critical parameter for biomolecules delivery systems obtained by electrostatic interactions that needs to be investigated because one gains information for possible effects on the mass, size and structural and colloidal stability at close to physiological conditions (C_NacL_ = 0.15 M simulates the concentration of the human blood). When the ionic strength increases in solutions that contain electrostatic complexes formed by polyelectrolytes and proteins, complex disintegration may occur. Such behavior originates from the decrease of electrostatic interactions between the oppositely charged groups because of salt screening effects. Alternatively, further aggregation or precipitation phenomena of complexes dispersed in the medium may occur due to salting out effects (decrease of solvent quality towards the dispersed complexes) [25,26].

In Figure 6, the effect of the increase of the ionic strength on the scattering intensity and R_h_ is presented. It is observed that during the additions of salt in the solution, the scattering intensity and the R_h_ values follow the same trend. During the first thee additions (i.e., up to C_NaCl_~0.06 M), a slight increase in the scattering intensity, which is accompanied with a more noticeable increase in R_h_ values, is observed. Based on the combination of the two factors, small salt concentrations result in further aggregation of the complexes, forming aggregates of complexes. Afterwards, the scattered intensity and the R_h_ values remain rather constant until the solution ionic strength increases above 0.45 M, where the intensity and R_h_ increase abruptly. It is of major importance that no significant decrease in the intensity occurs throughout the salt additions, because that would mean that the mass of the complexes would be decreased due to decomposition of the complexes. Until the salt concentration reaches ~ 0.45 M, it seems that an increase in the salt concentration does not affect the complexes until the last two salt additions, where the intensity and the R_h_ values increase again, indicating a second more severe aggregation of the complexes. Subsequently, it can be concluded that the QP(DMAEMA-co-LMA)-b-POEGMA/insulin complexes at the (−)INS/(+)polymer ratio equal to 0.25 remain rather stable and do not decompose, at the presence of salt, until the salt concentration becomes ~0.45 M which is considerably higher than the normal salt levels in human blood (~0.15 M).

**Figure 7:**
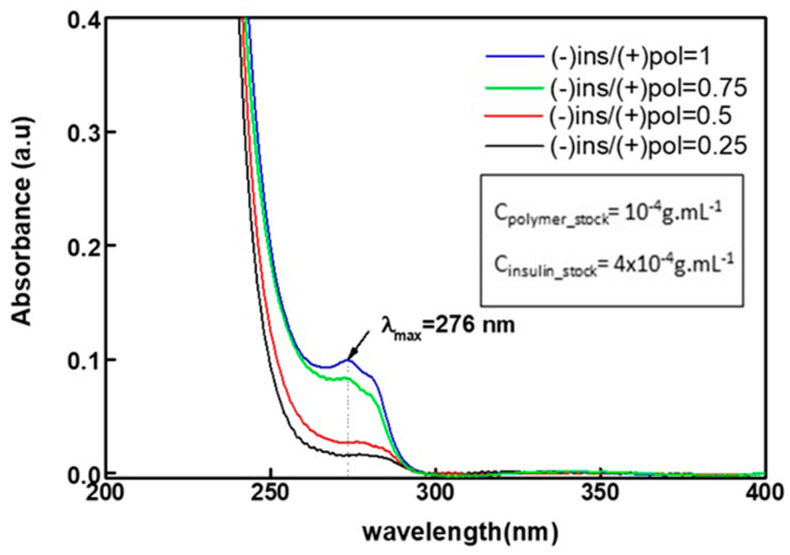
UV-Vis spectra of the QP(DMAEMA-co-LMA)-b-POEGMA/insulin complexes when the concentration of the polymer stock solution is 10*^−^*^4^ g·mL*^−^*^1^ and insulin stock solution is 4 × 10*^−^*^4^ g·mL*^−^*^1^.

UV studies were performed for the QP(DMAEMA-*co*-LMA)-*b*-POEGMA/insulin complexes in order to detect if structural changes occurred to insulin after the complexation with cationic polymer segments. Insulin consists of two peptide chains referred to as the A chain and B chain. A and B chains are connected with two disulfide bonds, and an additional disulfide is formed within the A chain [27]. Usually, the A chain consists of 21 amino acids and the B chain of 30 amino acids [28]. Tyrosine and phenylalanine residues of protein chains present optical absorption within the range of 230–300 nm [29]. UV studies in that wavelength range enable the determination of protein concentration, provide information about the local environment of these residues [30]. UV absorption in the range 180 to 230 nm is due almost entirely to π→π∗π→π∗ transitions in the peptide bonds, giving information about the protein secondary structure (protein folding) [29]. Unfortunately, insulin does not contain tryptophane, the more active amino acid residue in terms of fluorescence efficiency. However, tyrosine and phenylalanine residues of insulin can be utilized for UV-Vis studies. A considerable difference in the absorbance intensity is observed when (−)INS/(+)polymer charge ratio is 0.75 and 1 due to the presence of more insulin molecules in the mixed solutions (Figure 7). In these two cases, the peak of maximum absorbance wavelength is deciphered more clearly. The maximum absorbance wavelength (λ_max_) is observed at 276 nm. The λ_max_ is observed at the same wavelength in cases the (−)INS/(+)polymer charge ratio is 0.25 and 0.5 but the absorbance intensity is significantly lower. Based on literature data, the presence of a single peak at ca. 280 nm verifies that significant changes in the structure of insulin did not occur during the complexation process [28]. In other words, based on the UV-Vis spectra of the QP(DMAEMA-*co*-LMA)-*b*-POEGMA/insulin complexes, it is concluded that insulin did not undergo structural changes and is not denatured in all (−)INS/(+)polymer ratios investigated.

**Figure 8:**
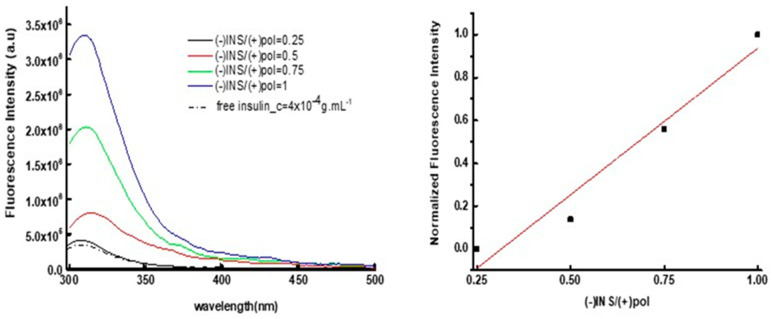
(**a**) Fluorescence spectra for QP(DMAEMA-co-LMA)-b-POEGMA/insulin complexes and insulin in aqueous medium (**b**) Normalized fluorescence intensity at 310 nm (peak maximum) as a function of (−)INS/(+)polymer charge ratio.

Fluorescence measurements were also conducted at excitation wavelength of 280 nm in order to provide more information on the conformation of insulin after its complexation with the terpolymer aggregates, via examining the intrinsic fluorescence of the amino acid tyrosine that is present in both insulin chains. The fluorescence spectra of the QP(DMAEMA-*co*-LMA)-*b*-POEGMA/insulin complexes at all (−)INS/(+)polymer charge ratios are depicted in Figure 8a and the normalized fluorescence intensity at peak maximum plot as a function of (−)INS/(+)polymer charge ratio at 310 nm (peak maximum wavelength) is displayed in Figure 8b. The variation of the wavelength in which the maximum insulin fluorescence intensity is noticed is less than 5 nm, indicating that there are no considerable differences in the conformation of the insulin after the QP(DMAEMA-*co*-LMA)-*b*-POEGMA/insulin complexes were formed. As it is expected, the fluorescence intensity rises as (−)INS/(+)polymer charge ratio increases. A linear trend is observed in the increase of the intensity as a function of (−)INS/(+)polymer charge ratio, which may only indicate that the increase of insulin concentration did not result in fluorescence quenching and/or precipitation of formed complexes.

**Figure 9:**
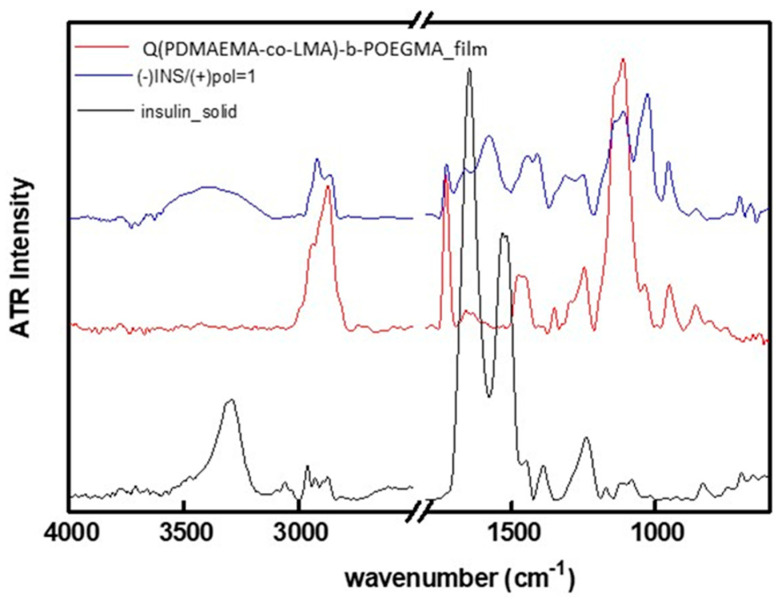
Comparative ATR-FTIR spectra of the solid insulin (black), QP(DMAEMA-co-LMA)-b-POEGMA deposited from aqueous solution after drying (red) and QP(DMAEMA-co-LMA)-b-POEGMA/insulin complex at (−)INS/(+)polymer = 1, after deposition and drying (blue).

ATR-FTIR measurements were conducted in order to investigate the interactions between the QP(DMAEMA-*co*-LMA)-*b*-POEGMA aggregates with insulin molecules at the functional groups level. In Figure 9, comparative ATR-FTIR spectra of solid insulin, QP(DMAEMA-*co*-LMA)-*b*-POEGMA film and QP(DMAEMA-*co*-LMA)-*b*-POEGMA/insulin complex at (−)INS/(+)polymer = 1 film, are presented.

Firstly, concerning the ATR-FTIR spectrum of solid insulin, the characteristic absorption band at 1665 cm^−1^ is assigned to amide I group and the one at 1532 cm^−1^ is assigned to amide II group mostly due to C=O stretching vibration typical of a protein spectrum. Regarding the ATR-FTIR spectrum of the QP(DMAEMA-*co*-LMA)-*b*-POEGMA film, the characteristic bands at 3003 cm^−1^, 1473 cm^−1^ and 948 cm^−1^ are attributed to the C-N(CH_3_)_3_^+^ group bond stretching vibrations of the quaternary amine, the absorption band at 1724 cm^−1^ is assigned to stretching vibration of the C=O carbonyl group, the one at 1144 cm^−1^ to the stretching of -C-N group, the one at 1126 cm^−1^ to the stretching of O=C-O-C ester group and the one at 1100 cm^−1^ corresponds to the stretching of C-O-C ether group. However, in the spectrum of QP(DMAEMA-*co*-LMA)-*b*-POEGMA/insulin complex, the two characteristic absorption peaks of insulin were almost concealed and only the peak assigned to amide I is still present but it is of very low intensity and rather shifted to smaller wavenumbers, compared with the one appearing in the ATR-FTIR spectrum of solid insulin. Moreover, no new peaks are observed in the ATR-FTIR spectrum of QP(DMAEMA-*co*-LMA)-*b*-POEGMA/insulin complex (Figure 9). These observations may indicate that some weak physical interactions between insulin and the QP(DMAEMA-*co*-LMA)-*b*-POEGMA aggregates were formed during the preparation of the complexes.

Skandalis et al. reported on the complexation of QPDMAEMA-*b*-PLMA-*b*-POEGMA triblock terpolymers with insulin [17]. Even though the same monomers were used, our polymeric system demonstrates different macromolecular architecture that has not been used before for complexation with insulin. QPDMAEMA-*b*-PLMA-*b*-POEGMA triblock terpolymers when inserted in aqueous media form spherical micelles, consisted of PLMA core and mixed QPDMAEMA/POEGMA corona [17], which can interact with insulin due to positive charge of the QPDMAEMA chain. On the other hand, when QP(DMAEMA-*co*-LMA)-*b*-POEGMA random-diblock terpolymers self-assemble in aqueous media, formation of nanoaggregates of spherical geometry is anticipated, due to the presence of the hydrophobic LMA component. The inner core of the nanoaggregates is expected to be of mixed nature and formed by QP(DMAEMA-co-LMA) random blocks, LS measurements indicate (R_g_/R_ho_ ratio in the range 0.6–0.68, Table 1). In the present case insulin is expected to reside within the inner part of the aggregates. The POEGMA hydrophilic blocks should make up the corona and offer stability to the formed aggregates, as well as more protection to the complexed insulin (e.g., towards enzymatic hydrolysis). Based on the different morphology of the nanoaggregates, complexes with insulin of different structural characteristics are also expected in the present case. This assumption is confirmed by the investigations reported on both research cases. Skandalis et al. used two QPDMAEMA-b-PLMA-b-POEGMA triblock terpolymers, which differ on the composition. Based on the results of their investigation, they concluded that the triblock terpolymer/insulin complexes are more stable and exhibit a higher size as insulin concentration increases and that the conformation of insulin stays intact during the complexation [17]. In our study, the physicochemical characteristics of the diblock terpolymer/insulin complexes depend on the concentration of the stock polymer and insulin solutions, as well as the diblock terpolymer/insulin charge ratio. In the case of the more concentrated stock solutions, the formed complexes were colloidally stable for three days, while when the less concentrated ones were used, the resulted complexes remained colloidally stable for weeks. The conformation of insulin was not affected by complexation, as was the case for the triblock terpolymer complexes [17]. Kamenova et al. also reported on the complexation of insulin with mixed block copolymer micelles. Specifically, they achieved the formation of mixed block copolymer micelles by mixing PEO-*b*-PCL-*b*-PEO and PDMAEMA-*b*-PCL-*b*-PDMAEMA triblock copolymers at appropriate molar ratios. The characteristics of the formed complexes depend on the mixed block copolymers micelles composition and insulin concentration [18], but also in this case insulin is interacting with the micellar corona and its protection by the PEO chains may be less effective.

## 4. Conclusions

In this work, the complexation ability of the QP(DMAEMA-*co*-LMA)-*b*-POEGMA aggregates with insulin molecules, towards formation of polymer/protein complexes was confirmed.

In vitro cytotoxicity experiments and histological tests have shown that the terpolymer utilized for the formation of complexes is in fact biocompatible and acceptable to be used as an insulin nanocarrier. The complexation takes place via electrostatic interactions between the positive charges of the cationic QDMAEMA segments and the negative charges of insulin chains. The complexes are stable at physiological conditions (neutral pH and 0.15 M NaCl). Size, mass, and surface charge of the complexes depend on block terpolymer/insulin charge ratio as light scattering techniques indicate. Based on the monomodal size distribution acquired by DLS, only one type of population was present in the aqueous solutions of the formed complexes. It was observed that when the stock solutions of lower concentration were used, for both polymer and protein components, complexes of lower mass but of higher size were formed. Surface charge of the complexes was positive and showed no significant difference, in all ratios utilized, indicating that the cationic QPDMAEMA segments occupy the periphery of the complexes, hiding the negatively charged insulin molecules. Precipitation phenomena were observed three days after complex formation when higher concentrations of the components were used, indicating that the colloidal stability is affected by the concentration of the stock solutions utilized.

UV-Vis and fluorescence spectroscopy studies confirmed that no structural alterations occur at the insulin conformation, after complexation.

Based on the results presented QP(DMAEMA-*co*-LMA)-*b*-POEGMA aggregates comprise promising insulin nanocarriers that may be useful for insulin delivery applications in vivo.

## Figures and Tables

**Table 1:** Molecular and self-assembly characteristics of the P(DMAEMA-co-LMA)-b-POEGMA and QP(DMAEMA-co-LMA)-b-POEGMA diblock terpolymers.

Sample	M_w_^a^ (× 10^4^) (g/mol)	M_w_/M_n_^a^	% wt. PDMAEMA ^b^	% wt. QDMAEMA ^b^	% wt. PLMA ^b^	% wt. POEGMA ^b^	Intensity ^c^ (a.u)	R_h_^c^ (nm)	R_g_/R_h0_^e^	ζ_p_^d^ (mV)
P(DMAEMA-*co*-LMA)-*b*-POEGMA	1.3	1.25	12	-	7	80	50	2.23/150	0.68	+2.5
Q(PDMAEMA-*co*-LMA)-*b*-POEGMA	1.46	-	-	22	6	72	250	107	0.6	+37

^a^ Determined by SEC. ^b^ Determined by ^1^H-NMR. ^c^ Determined by DLS at 90°. ^d^ Determined by ELS. ^e^ Determined by a combination of DLS and SLS, after extrapolation to zero angle.
